# Development of a Rice Plant Disease Classification Model in Big Data Environment

**DOI:** 10.3390/bioengineering9120758

**Published:** 2022-12-02

**Authors:** Shampa Sengupta, Abhijit Dutta, Shaimaa A. M. Abdelmohsen, Haifa A. Alyousef, Mohammad Rahimi-Gorji

**Affiliations:** 1 Department of Information Technology, MCKV Institute of Engineering, Liluah, Howrah 711204, India; 2 Department of Mechanical Engineering, MCKV Institute of Engineering, Liluah, Howrah 711204, India; 3 Department of Physics, College of Science, Princess Nourah bint Abdulrahman University, P.O. Box 84428, Riyadh 11671, Saudi Arabia; 4 Faculty of Medicine and Health Sciences, Ghent University, 9000 Ghent, Belgium

**Keywords:** data mining, big data, rough set theory, ensemble classification, rice disease prediction

## Abstract

More than the half of the global population consume rice as their primary energy source. Therefore, this work focused on the development of a prediction model to minimize agricultural loss in the paddy field. Initially, rice plant diseases, along with their images, were captured. Then, a big data framework was used to encounter a large dataset. In this work, at first, feature extraction process is applied on the data and after that feature selection is also applied to obtain the reduced data with important features which is used as the input to the classification model. For the rice disease datasets, features based on color, shape, position, and texture are extracted from the infected rice plant images and a rough set theory-based feature selection method is used for the feature selection job. For the classification task, ensemble classification methods have been implemented in a map reduce framework for the development of the efficient disease prediction model. The results on the collected disease data show the efficiency of the proposed model.

## 1. Introduction

Agricultural data contain a large volume of data with large variety and veracity too. Data velocity is also very high since new data are being generated frequently from every application and added to the existing data. Handling of these kinds of complex data is a very challenging task in the field of data mining. The data are not always structured, rather, there is a collection of structured, semi structured, and unstructured, too. To analyze these data, at first, proper structured must be given with the help of different IT tools and techniques. Thus, before applying any algorithm proper preprocessing stage is necessary. In the following Section 1.1, different types of rice diseases along with their symptoms are discussed.

### 1.1. Rice Diseases and Their Symptoms

In this section, different rice diseases [1] related to our work and the various symptoms of the diseases are discussed.

1. Rice leaf blast: elliptical to oval spots generally light colored in centers and edges are dark reddish-brown.
2. Brown spot: Fungal infection causes round to oval dark brown color spot.
3. Sheath blight: Irregular or oval with greenish grey spots. When the spots become enlarged, the center becomes greyish white and the margin turns a blackish-brown color.
4. Bacterial leaf blight: yellowish lesion with uneven edges. Leaves become yellow and gradually die.
5. Sheath rot: Spots are irregular with greyish white in the center and a brown margin. There is discoloration of the seed.

Now, to develop a disease prediction model, the following steps are applied on the diseased images [2].

(i) Image acquisition: In this step, the images are captured from the field directly through different devices such as camera, mobile phone, etc.
(ii) Image preprocessing: In this stage, preprocessing is performed on the infected images to eliminate the unwanted noises, such as water droplets, and image enhancement is also applied by some standard techniques.
(iii) Image segmentation: In this step, the region of interest on the image being identified with different segmentation technique such as k-means clustering, Otsu’s threshold method, Pixel-based, Fermi Energy, Fractal Descriptors, and watershed methods are used [3,4].
(iv) Feature extraction and selection: In this step [5], at first, important features are generated from the diseased object and then important features are selected from the feature pool. Different feature extraction techniques are used to extract the features related to statistical, color, shape, texture, wavelets, size, area, proximity, and centroids, morphology features, correlation-based feature, textural descriptors using GLCM [2] and color moments, etc. Different soft computing-based feature selection techniques such as rough set theory [6,7], Genetic Algorithm [8], etc. are used to select the important features as well.
(v) Classification: The final step decides the class label of the disease through different classification techniques. Various state-of-the-art classification tools and techniques exist, such as SVM [9], artificial neural network [10], decision tree [11], k-NN [11], and rule-based techniques, etc., to perform the classification task.

For the rice plant disease data, preprocessing tasks such as feature extraction and feature selection are a very important step to gain insights from the data. Various feature extraction techniques [12] are used to generate the important features from the dataset. As large features are being associated with the image data so feature selection [13] is also necessary to get the efficient prediction model. The feature selection process selects the important features before the classification task to reduce the space and time complexity of the model.

Deep learning has been used in image classification problems recently in a large manner, still, it can be seen that the deep learning model does not have that much of efficiency for small-size data and at the same time, it is not easily interpretable how things work inside the deep network. The proposed method has a proper understanding of all the stages with an emphasis on feature extraction and feature selection stages where the important features are clearly visible.

**Figure 1:**
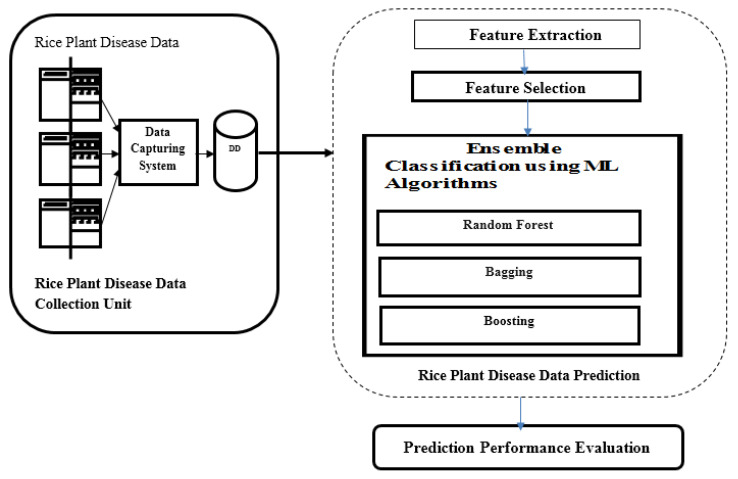
Proposed Overview of the Model.

**Figure 2:**
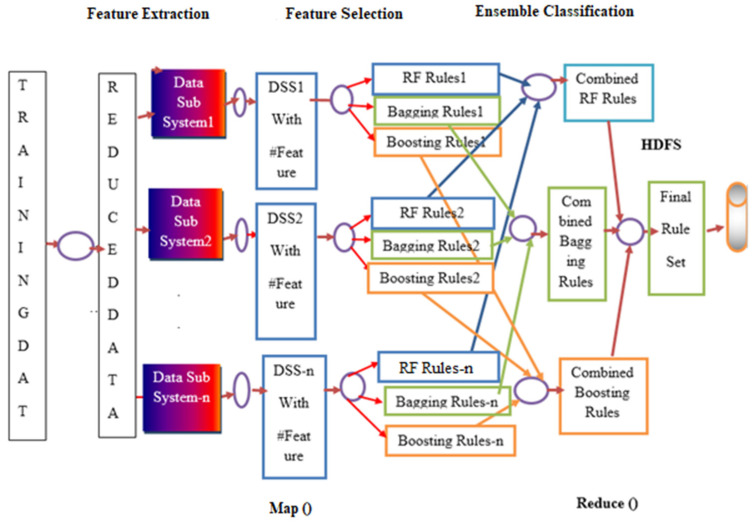
Proposed Big Data-Enabled detailed Disease Prediction Model.

In the paper, an integrated environment is proposed for the prediction of the rice plant disease in a big data environment. In the first step, the different features related to color, shape, position, and texture are extracted [14] from the infected rice plant images. Then, in step two, a rough set theory-based feature selection algorithm is applied on the reduced data to generate the most important features to reduce the model overall complexity. In step three, ensemble classification methods are used to predict the disease. The whole system was implemented in the map Reduce framework of Hadoop platform [15]. The overall workflow of the proposed method is presented in Figure 1. Detailed MapReduce framework implementation is presented in Figure 2.

The primary contribution of the work is:

- Analyzing rice plant disease data using a machine learning algorithm in the MapReduce framework of a big data platform;
- Selecting multiple disease feature subsets using a rough set theory-based feature selection algorithm;
- Developing integrated feature extraction, feature selection, and ensemble classification techniques in a map reduce framework for better disease prediction results.

In the paper, Section 2 describes the issues of big data in prediction of rice plant diseases, whereas Section 3 describes the development of the expert rice plant disease prediction model. Section 4 presents the experimental results. Section 5 presents the salient features of the method with the conclusion and future scope.

## 2. Big Data in Plant Disease Prediction

Since, nowadays, data are being generated at an alarming rate, a big data framework is necessary to analyze these datasets properly. The speed of data generation is very high and at the same time, the volume of the data is huge too. The type of data also varies in nature. Every such as like science, engineering, business, and healthcare is generating these big data. Thus, to handle these types of data, a big data platform is necessary where data can be analyzed efficiently to generate the value from the raw data to perform any kind of decision-making task. Different big data processing tools are available which researchers can use for their research purposes. Hadoop [15] is a framework where the data can be stored and processed. HDFS actually stores the data and map reduce is the main processing unit of the Hadoop system. Fast and parallel processing can be achieved here with the master slave fashion distributed as it is processed.

Agricultural data are mostly unstructured as they are generated from the plant image data. The data size is also huge as lots of image data are being collected nowadays globally. Thus, to process these data, at first, the unstructured data are converted to a structured one and then data preprocessing tasks such as noise reduction, segmentation and data dimension reduction tasks are performed to obtain interesting patterns from the data. As the data size is also huge, dimension reduction is very much necessary to develop the model efficiently.

### 2.1. Ensemble Classification Techniques

Sometimes, a single classification system does not provide the accurate results where the combination of multiple classification system provides better results and is termed as an ensemble classification system. In this technique, a group of basic weak learners are combined to produce the strong ensemble learning system with some multiple decision trees combined to produce better predictive results in ensemble methods or learning [16]. These base learners may differ in their algorithm approaches, parameters, representations, or in the training sets.

An ensemble decision tree is built mainly by using two techniques:

Bagging: It is an ensemble classification method [11] where the output of the base learners is combined and final decision is taken based on that combined output. The method is also called bootstrap aggregator. A dataset is bootstrapped into a different sample dataset using row sampling with a replacement technique. For each and every sample dataset, an individual model is built by using some base learning technique. The base learning technique can be any classification technique. For the training dataset, using row sampling with a replacement technique, all base learning models will be trained and when a test data comes, the outputs of all the base learners are combined with any kind of voting concept to assign the class label of the test object.

Random Forest: It is an ensemble classification method [11] based on the bagging technique. To design an ensemble technique, the selection of base learner is very important. Here, we used the decision tree classifier as the base learners for the development of the prediction model.

Suppose there is a dataset *D* with *A* no. of conditional attributes/features represented in columns and *d* no. of records or objects represented in rows. To start with the process, at first through row sampling and a feature sampling process, a new dataset *d*_1_ is created and after applying the decision tree method on the dataset, the model M1 is created for the dataset *d*_1_. Then, again by row sampling and a feature sampling process, the new dataset *d*_2_ is created where repetitions of some records and features may be present and then, applying the decision tree method, model M2 is created for the dataset *d*_2_. In this way, all base models are developed. Then, by aggregating the output with a majority voting concept all the base models’ outputs are combined and a final decision is made for the class of the test object. Generally, when a decision tree is made with the complete depth on the training data, then a high variance problem may occur in the process of classification, but here, as the aggregation of the outputs is taken, high variance becomes low variance, which itself proves the advantage of using the Random Forest method.

Boosting: The method [11], combining the output of multiple weak learners through voting or weighted average method a strong learner is built to predict the output of the test object. Here, more weightage is given to the misclassified objects till the objects become classified correctly. Here, weak learners are developed by using the base machine learning technique such as decision tree by default on the different distributions of the dataset.

### 2.2. Previous Works on Rice Plant Disease Prediction

The proposed method does not involve any cost, but it gives a better prediction result by developing an expert system through a feature selection and ensemble classification approach in big data environment.

In this section, different recent works on rice disease classification are discussed. The work in [17] discusses the development of the rice disease detection model. Otsu’s segmentation method is used to extract the region of interest from the image where features are selected from LBP and classification is performed using SVM with three kernel functions, Linear, Polynomial, and Radial Basis Function (RBF). The paper in [18] proposes a rice blast disease classification model where the Watershed method is used for segmentation. Texture and shape features are used to classify the diseases with accuracy level of 94%. The paper in [19] proposes an improved k-NN along with k-means to classify the rice diseases. Otsu’s segmentation method is used to extract the region of interest. They use shape and color features which are used to classify the diseases. A classification accuracy of 94% was achieved using the developed model. Different parameters such as sensitivity and specificity were measured as well to show the effectiveness of the model. The paper in [20] proposes a system to detect bacterial blight, rice leaf blast, and brown spot rice diseases. They used a k-means clustering technique to separate the damage and the undamaged portion. Features such as color, size, centroids, and proximity are used to classify the diseases. The paper in [21] developed a system to classify rice blast, bacterial blight, and sheath blight diseases. Otsu’s method is used for the segmentation purpose where Combination of FCMKM and Faster R-CNN used for the detection of the rice diseases. Different accuracy levels for different diseases were presented, i.e., rice blast—96.71%, bacterial blight—97.53%, and blight—98.26%. The authors of [22] propose a prediction model for predicting leaf blast, brown spot, and leaf blight diseases in paddy leaf. Otsu’s method is used for the segmentation purpose. Features related to wavelets and textures are used to classify the diseases. A feed-forward neural network (FFNN) is used for the classification job. An average accuracy of 91% was achieved. The paper in [23] proposes a disease detection model using machine learning techniques such as k-NN, Naive Bayes, Logistic Regression, and Decision Tree. Feature extraction is done through a correlation-based extraction method. An average classification accuracy of 97% was achieved. The authors of [24] proposed a method for developing the disease detection model with three different stages of severity of the diseases such as infection, spreading, and worst stage. A multi-level thresholding method is used to create the segmentation task. Shape and color features are used to detect the severity level of the disease. The authors of [25] developed a model for the detection of rice leaf blast disease. Segmentation is achieved through a k-means clustering algorithm. Statistical and texture features are used for the classification of the severity level of the rice leaf blast diseases. ANN is used to perform the classification. Feature selection methods generate some extraneous features, as the method uses only a forward feature selection technique without backward elimination without any proper guidance for handling the big data either [13].

Many works exist on developing disease classification systems, but those systems are not big data-enabled, so our contribution is towards developing an efficient disease prediction model suitable for handling large data.

## 3. Development of Expert Rice Plant Disease Prediction Model

The objective of the proposed work is the development of an expert prediction system for rice plant diseases in a big data environment.

Agricultural data are vast and unstructured, so to analyze these types of datasets, a big data-enabled environment is needed. To predict the rice plant disease, a dataset containing the different disease features with their values and based on the feature value different class labels of the disease is assigned. We know features with maximum contribution to predict the disease are the important ones. To develop the efficient prediction model, important features are selected before the classification job. A single classifier’s output prediction on the disease is not always correct, so in that case, a multiple classifier associated model with majority voting principle may be the solution. Feature selection and classification are the two main processes for developing an efficient prediction model. The whole system then produces better results in terms of accuracy in disease prediction. The system is implemented in the big data environment to accommodate larger disease datasets in the agricultural domain.

In the proposed work, a disease prediction model is developed to predict the rice plant disease in the Hadoop platform [15]. Map reduce processes are used to implement the work. Feature selection and classification modules are mapped with map reduce processes and implemented in the big data platform. Feature subset selection is performed through a feature selection algorithm [26] based on rough set theory and in the classification phase, different ensemble classifiers play the base classification role and the final output of the classification system is achieved through majority voting principle. Adaboost [11], Random Forest and Bagging [11] with classifier are chosen as the base classifier. The detailed working of the proposed method is presented in the following subsections.

### 3.1. Feature Selection

Selecting features and classifying the object based on that feature is the main task of the developing model in map-reduce framework for disease prediction. This work comprises the rough set theory [based multiple feature selection method [26] for the selection task. A rough set-based multiple feature subset selection method is used for selecting multiple feature subsets from the dataset without losing any information. The advantage of using this method is that the method can handle real-valued data as well. The method is based on the concepts of indiscernibility relation of rough set theory [7,27,28], graph theory [29] and clustering algorithm [30]. The novelty of the method is that the big data mining problem is converted to a graph theoretic problem and then a multiple feature subset is generated. The method has a strong mathematical foundation, so it produces better results in terms of selecting features.

The feature selection method was developed by using the concept of rough set theory, graph theory and clustering algorithm. A simple k-means clustering algorithm is used for the continuous-valued dataset, whereas the K-protoype clustering algorithm is used for the categorical dataset for the clustering purpose. The steps of the method are given below.

Let *DS =* (*U, F*) be a decision system where *U* is the finite, non-empty set of objects and *F = A* ∪ *D* such that *A* and *D* are a set of conditions and decision attributes, respectively.

I. Partitioning the Objects of the decision system

The objects are partitioned in two different ways:

(a). Partitioning of objects based on decision attribute using indiscernibility relation

For decision attribute *D*, the equivalence classes are *U*/*D* obtained by *IND (D)* using the indiscernibility relation

(1) $$\mathrm{Let}\;U/D=CL^{D}=\{CL_{1}^{D},\;CL_{2}^{D},\;\ldots \ldots \ldots ,\;CL_{k}^{D}\}$$

(b). Partitioning of objects by applying clustering algorithm on the projections of the dataset.

Let *A* = {*A*_1_, *A*_2_, ……, *A_n_*} be the set of conditional attributes. Now, projection on the dataset *DS* for two attributes *A_i_* and *A_j_* is performed to obtain the projected dataset (*PDS*).

(2) $$PDS=\prod_{A_{i},A_{j}}\left(DS\right)$$

Therefore, *PDS* contains the same number of objects as *DS*. Now, the dataset *PDS* is clustered using a k-means or *K*-prototype algorithm with *K* as the number of distinct values of decision attribute *D*.

Let the clusters obtained by *A_i_* and *A_j_* be

(3) $$CL^{ij}=\{CL_{1}^{ij},\;CL_{2}^{ij},\;\ldots \ldots \;CL_{k}^{ij}\}\mathrm{for}\;\mathrm{all}\;i,j=1,2\ldots ,m;\;\;i<j.$$

II. Computation of attribute connecting strength

Here, computation of connecting strength between attributes *A_i_* and *A_j_* is made based on those two partitions obtained from the above Equations (1) and (3). Then, the connecting power or connecting factor of the attributes *A_i_* and *A_j_* is measured and denoted by $\delta_{f}^{i,j}$ using Equation (4). The connecting factor measures the degree of connectivity of the features/attributes among each other concerning decision attributes/features.

(4) $$\delta_{f}^{i,j}=\frac{1}{K}\sum_{CL_{t}^{ij}\in CL^{ij}}\frac{1}{CL_{t}^{ij}}\underset{\forall \;CL_{P}^{D}\in CL^{D}}{\max}\left\{CL_{t}^{ij}\cap^{\;}CL_{P}^{D}\right\}$$

Therefore, $\delta_{f}^{i,j}=1;\;$ if *A_i_* and *A_j_* are totally connected with respect to *D* < 1.

Let the attribute connecting set *ACS* = {$A_{i}A_{j}\overset{\delta_{f}^{i,\;j}}{\to }D$ ∀ *i*, *j*} which consists of all possible pairwise connections of attributes. Now, the average connecting factor *δ_f_* is computed and the elements $A_{i}A_{j}\overset{\delta_{f}^{i,j}}{\to }D$ with $\delta_{f}^{i,j}<$δ*_f_* are discarded, and the rest is considered as the modified attribute connecting set *MCS*.

III. Construction of attribute connecting graph

Now, from the *MCS*, a weighted undirected graph *ACG* = (*V*, *E*) is constructed as follows:

- For each element $A_{i}A_{j}\overset{\delta_{f}^{i,\;j}}{\to }D\in MCS$:
  (i) *A_i_* and *A_j_* are considered as vertices of the graph *G,* i.e., $V=V\cup \{A_{i}\}\cup \{A_{j}\}$ where *V* = {∅} initially;
  (ii) An edge (*A_i_, A_j_*) is drawn with weight $\delta_{f}^{i,j}$, i.e., $E=E\cup \{(A_{i},\;A_{j})\}$ where *E* = {∅} initially. Thus, *E* is a proper subset of *V × V*.

This graph is called the attribute connecting graph *ACG,* which represents how the attributes are connected to represent a decision system.

IV. Generation of Reduct

The undirected weighted graph *ACG = (V, E)* has weighted edges. The weight of an edge indicates the classification power of the attributes corresponding to the terminal nodes of the edge. The higher the weight of an edge, the better the classification power of the combined attributes (nodes). Now, a term degree of connection of a node is defined as follows:

Let *ACG = (V, E)* be an undirected weighted graph and *n_i_* ∈ *N* be a node. Then, the degree of connection of a node *n_i_* denoted by *dc*(*n_i_*) is defined as

(5) $$dc(n_{i})=\frac{1}{deg\left(ni\right)}\sum^{\;}w_{ij}\;/(n_{i},n_{j})\;\in \;E\;\mathrm{and}\;w_{ij}\mathrm{is}\;\mathrm{the}\;\mathrm{weight}\;\mathrm{of}\;(n_{i},n_{j})$$

where deg *(n_i_*) is the degree [29] of the vertex *n_i_*_._

Here, the higher the degree of connection, the more important the corresponding attributes. Initially, the highest degree node *v* associated with the attribute is considered as the reduct and *v* is removed from the attribute connecting graph (*ACG*) accordingly. Therefore, the ‘degree of connection’ of the vertices incident on the removed vertex is reduced by the weight associated with the corresponding edge. Thus, the graph *ACG* is modified, and the new attribute associated with the current highest degree of connection is added to the reduct and repeats the same process until all the edges are removed or the graph becomes empty. Here, multiple reducts or multiple feature subsets will be generated if more than one vertex has the highest degree of connection at some iteration.

Details are given in the paper in [26]. Here, an illustration of the method is given for a better understanding of the proposed method.

V. Illustration of the feature subset selection method:

Let a decision system *DS* consist of 8 objects {*O*_1_, *O*_2_, *O*_3_, *O*_4_, *O*_5_, *O*_6_, *O*_7_, *O*_8_} and 4 conditional features {*C*_1_, *C*_2_, *C*_3_, *C*_4_} and one decision feature *D* with 2 decision classes.

(i) At first, *DS* is partitioned based on *D* using indiscernibility relation and allowing the following equivalence classes to be obtained:

$$\;CL_{1}^{D}=\{O_{2},\;O_{3},\;O_{4},\;O_{5}\}\;\mathrm{and}\;CL_{2}^{D}=\{O_{1},\;O_{6},\;O_{7},\;O_{8}\}$$

(ii) Projection on dataset *DS* for two features *C*_1_ and *C*_2_ is taken and k-means clustering algorithm is applied on it with *k* = 2 that produces following two clusters:

$$\;CL_{1}^{12}=\{O_{2},\;O_{3},\;O_{4},\;O_{8}\}\;\mathrm{and}\;CL_{2}^{12}=\{O_{1},\;O_{5},\;O_{6},\;O_{7}\}$$

(iii) The connecting factor δ*_f_*^1,2^ for two attributes *C*_1_ and *C*_2 is_ calculated

$$\delta_{f}^{1,2}=\frac{1}{2}\left\{\frac{1}{4}\times 3+\frac{1}{4}\times 3\;\right\}\;=\;0.75$$

(iv) In this way, after applying the clustering algorithm on each pairwise feature in {*C*_1_, *C*_2_, *C*_3_, *C*_4_}, a feature connecting set (*FCS*) representing the connection of every pair of conditional features to the decision feature is constructed.

$$ACS=\{C_{1}C_{2}\overset{0.75}{\to }D,C_{1}C_{3}\overset{0.82}{\to }D,\;\;C_{1}C_{4}\overset{0.85}{\to }D,\;\;C_{2}C_{3}\overset{0.88}{\to }D,C_{2}C_{4}\overset{0.90}{\to }D,\;C_{3}C_{4}\overset{0.73}{\to }D\}$$

(v) The elements of *FCS* with a connecting factor less than the average value are removed, and a modified *FCS* is formed.

**Figure 3:**
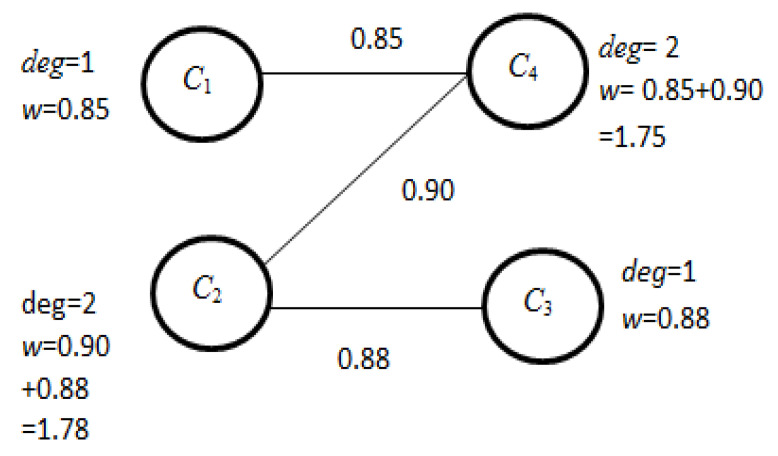
*FCG* achieved from modified *FCS*.

**Figure 4:**
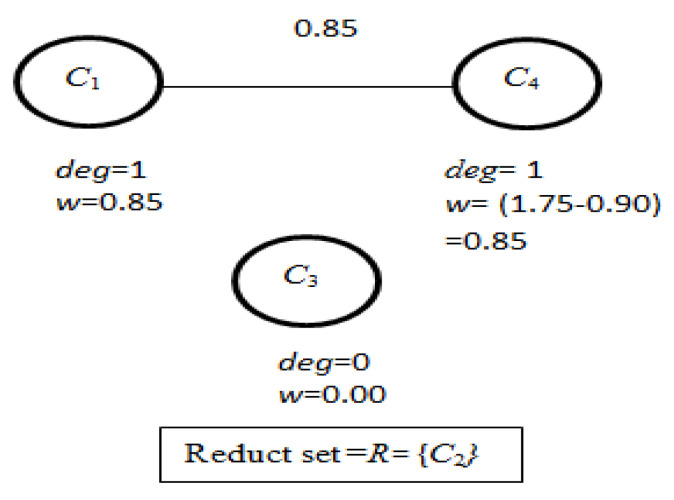
Modified *FCG* after first iteration.

**Figure 5:**
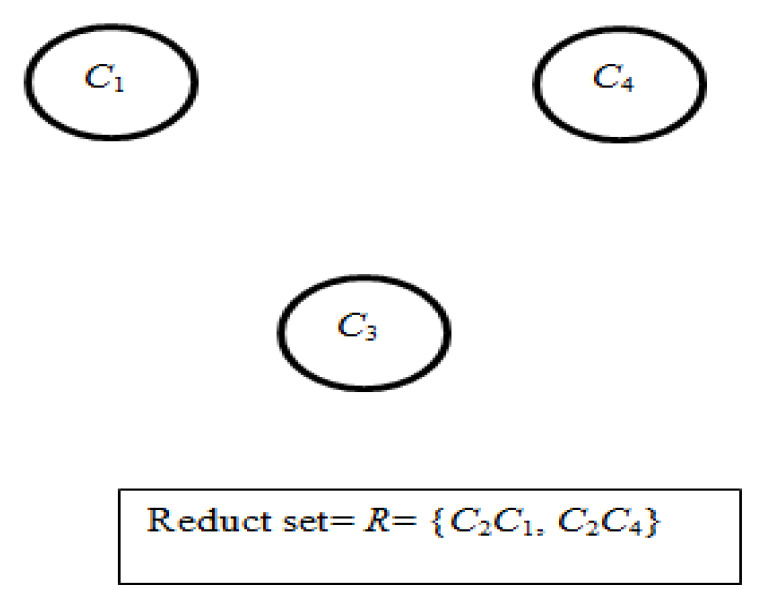
Modified *FCG* after second iteration.

Here, the average connecting factor (δ*_f_* ) = 0.83. Therefore, the modified *FCS* is

$$FCS=\{C_{1}C_{4}\overset{0.85}{\to }D,\;\;C_{2}C_{3}\overset{0.88}{\to }D,\;C_{2}C_{4}\overset{0.90}{\to }D\}$$

(vi) Now, a feature connecting graph (*FCG*) is formed from modified *FCS*. Figure 3 represents the attribute connecting graph for the example data.

(vii) Now, from *FCG,* the *degrees of connection* of each vertex *v* (here it is *C*_1_, *C*_2_, *C*_3_, and *C*_4_) are calculated.
(viii) From *FCG*, the vertex *C*_2_ has the highest degree of connection and according to the ‘Multiple_Reduct_Gen’ algorithm, *C*_2_ is considered as the reduct *R* and removed from *ACG* with the adjustment of degree of connection of the vertices adjacent to it. Figure 4 represents the modified *FCG*.

(ix) Now, in the next iteration, two vertices *C*_1_ and *C*_4_ have the same *degree of connection* and so, according to the ‘Multiple_Reduct_Gen’ algorithm, for a single reduct in previous iteration and 2-vertices of highest degree of connection in *FCG*, 2 reducts are obtained. This process provides two reducts at the end of this iteration with *R* = {*C*_2_*C*_1_ and *C*_2_*C*_4_} and *FCG* becomes empty, as shown in Figure 5, which indicates the termination of the iteration.

### 3.2. Multi Classifier-Based Classification Module

For efficient classification, we use an efficient multiple feature subset selection algorithm based on rough set theory [7,27,28] with ML Algorithms such as Random Forest, Bagging [11], and AdaboostM1 Algorithm [11] in the MapReduce framework computation [15]. A majority voting technique is used to make the final decision on the disease in the model. The detail implementation of the algorithm in the Map Reduce framework is described in the next section.

Map Reduce framework implementation:

Initial data from HDFS [15] are collected. The whole process works in the following manner: Select multiple feature subset for each of the decision subsystems and then generate classification rules for each of the decision subsystems using base classifiers. Final prediction on the class label is achieved through majority voting on the base classification rules.

The whole method has three phases—Map phase, combine phase, and Reduce phase.

A. Map phase—In this phase, the Map function applies a feature selection algorithm on each of the decision subsystems and computes the individual feature subset for each decision subsystem, and then applies the base classifier on each of the reduced subsystems to generate classification rules and the output of the map phase is the input to the combine phase.

B. Combine phase—The Combine phase has its own combination function and the inputs of the function are the output of the previous Map phase. This phase produces the classification rules for all the classes for each of the decision subsystems and, finally, results are sent to the Reducer function.

C. Reduce phase—In this phase, the Reducer function works on the output of the combiner phase and then majority voting is applied to obtain the final classification rules for the whole dataset. Finally, classification rule sets are stored in HDFS.

The overall proposed algorithm is given below in Algorithm 1:
**Algorithm 1.** Classification Model Generation 1. Initialize a training Dataset *d* = {(*_Oi_*, *c_i_*), *i* = 1, 2, ………, *n*}, where labels *c_i_* is one of 1, 2, 3, ………, *k*, A set of base classifiers *B* = {*b*_1_, *b*_2_, ………, *bt*} and *N* = no of nodes2. Apply feature extraction method on *d* to obtain the preprocessed reduced dataset *DSS*3. Split *DSS* into *DSS*_1_, *DSS*_2_, …, *DSS_N_* and map into the corresponding node4. for each node *i* = 1 to *N*5.   Apply feature selection method to get the reduced data subsystem *RDS_i_*6.    for each base classifier *b_t_* in *B*7.     train *b_t_* on *RDS_i_* and compute classification rules in *Rt*8.    end for9. end for10. for each node *i* = 1 to *N*11.   for each *b_t_*12.    Combine all *R_t_* of *RDS_i_* and Create rule base *CR_k_* for each *k*13.   end for14. end for15. for each test object16.   Apply majority voting rule on *CR_k_* to get the label of the test object17. end for

For a dataset with N no. of data objects with D dimension, for ensemble methods Random Forest and Bagging, the training time complexity for both the algorithms is O (K N logN D) where K is the number of trees. For Boosting algorithms, the training time complexity of the algorithm is O (K N logN D), but practically, it takes more space than Bagging as the previous tree’s error values have to be retained for generating the next level tree. Therefore, the complexity of the model developed using the ensemble algorithms in an aggregation manner is slightly high, but as it is applied in the big data environment then at the same time, the overall veracity component of the model is reduced.

## 4. Experimental Results

**Figure 6:**
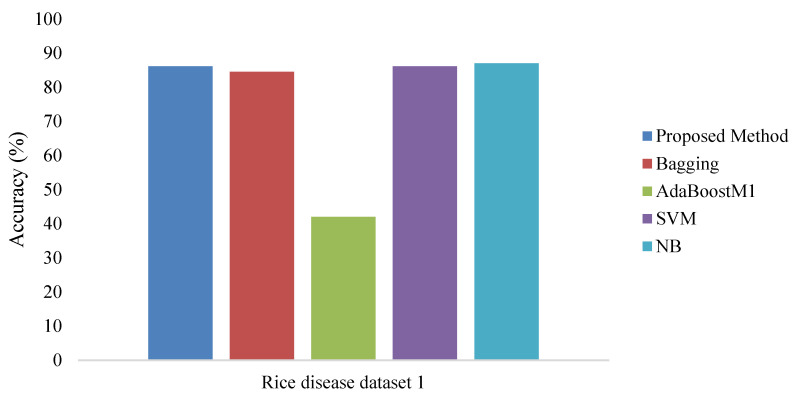
Accuracy for Rice disease dataset1 in the big data environment.

**Figure 7:**
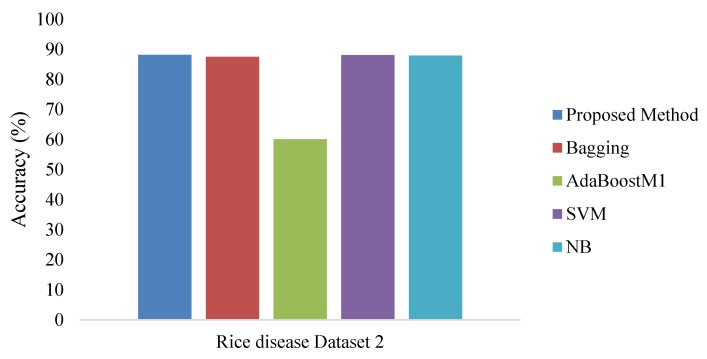
Accuracy for Rice disease dataset2 in the big data environment.

To measure the performance of the proposed method, rice disease image data1 [5,13] and rice image data2 were considered for analysis as mentioned in [31,32]. The algorithm is implemented using Python language in the cloud-based Hadoop platform. The considered rice disease dataset [13] contains data with four rice plant disease classes—rice blast, sheath rot, leaf brown spot, and bacterial Blight. Five hundred infected rice plant images were considered with 25 features. The shape and texture features [13] were considered to classify the diseases. After selecting the 16 important features, they were considered for the classification. Shape features include spot, area, perimeter, area discrepancy, aspect ratio and momentums (U1–U6) of the infected region. The model accuracy was calculated and compared with different state-of-the-art classification methods to review the efficiency of the proposed method. The existing methods run in Weka [32]. Here, a k-fold cross validation method was used to build the model where k is the number of fold/partition and the value of k was fixed as 10 for our method. All the results on Rice disease dataset1 and Rice disease dataset2 are provided in Figure 5, Figure 6 and Figure 7. The trio reported that the technique used for the proposed method is superior to other single classification technique and simple ensemble techniques. To judge the classifier performance fully, various classification parameters such as classification accuracy, precision, recall, etc. were evaluated through a series of experiments and the results are presented below in Table 1 and Table 2.

The above results prove that the proposed method is better and more efficient with respect to other standard methods. The proposed ensemble-based method in the big data framework performs well in feature selection, as well as being efficient in classification without losing too much information. The proposed method yields a better prediction result for detecting the rice disease.

## 5. Discussion and Conclusions

An expert disease prediction model for the agricultural domain was devised to predict the disease at early stage to save the plants. This intelligent model has the capacity to capture the diseased image from the beginning to classify the plant disease. The model is also applicable for analyzing big data. The method can handle any type of data, as also discussed. In the model, mainly feature selection and classification unit are integrated and implemented in the Map Reduce framework. In the Map Reduce process, in each node, at first RST-based feature selection method is used to select the important feature subsets, then Random Forest, Bagging and Adaboost techniques are applied to generate the classification rules for each reduced dataset. Then, output from all the reduced datasets is combined with the majority voting technique to generate the classification rule to classify the disease efficiently. Since the MapReduce approach is used in the proposed method, efficiency will not be an issue at all because data processing is performed in the different nodes. The classification model provides an accuracy level of about 88.19% for the rice disease dataset. The benefits of our method are: (i) providing multiple feature subsets using RST-based graph theory and clustering algorithm; (ii) the method can handle real valued data (iii) S. Phadikar et al. [13] feature selection methods to generate some extraneous features, as the method uses only a forward feature selection technique without backward elimination; (iv) the proposed algorithm can be applied in a big data environment, where the data volume is large.

The proposed method is efficient for classifying other plant diseases. This approach can be useful for handling large data of other fields such as social media, bioinformatics, ecommerce, etc. More experiments must be conducted on handling the incremental data in this big data platform to predict the diseases of the plant.

The proposed method is a generalized prediction method and provided good results to classify the human disease too. The proposed method has many benefits in terms of societal and technological aspects, still, more experiments should be conducted on the other agricultural datasets to prove the broader aspects of the method in a big data environment.

The performance of the model can be checked by incorporating the deep learning-based method instead of using traditional machine learning-based methods implemented in the big data platform.

## Figures and Tables

**Table 1:** Comparative Classification accuracy for other disease datasets.

Dataset (Original Attribute)	Bagging (%)	AdaBoost (%)	Proposed Method (%)
Breast Cancer (9)	94.43	95.74	96.93
Heart (12)	82.25	82.97	84.51
Dermatology (33)	96.01	96.26	98.99

**Table 2:** Comparative Classification values for other disease datasets.

Dataset (#Original Attribute)	Classification Methods	Classifiers Parameter Values		
		Precision	Recall	F-Measure
Breast Cancer (9)	Proposed Method	0.97	0.97	0.97
	Bagging	0.95	0.95	0.95
	AdaBoostM1	0.96	0.96	0.96
Heart (12)	Proposed Method	0.84	0.84	0.84
	Bagging	0.82	0.82	0.82
	AdaBoostM1	0.83	0.83	0.83
Dermatology (33)	Proposed Method	0.99	0.99	0.99
	Bagging	0.96	0.96	0.96
	AdaBoostM1	0.96	0.96	0.96

## Data Availability

All data generated or analyzed during this study are included in this article.
